# Compound Comminuted Fracture of Skull—Case

**Published:** 1874-07

**Authors:** R. Y. Rudicil

**Affiliations:** Trion, Ga.


### COMPOUND COMMINUTED FRACTURE OF SKULL—CASE.

By R. Y. Rudicil, M.D., of Trion, Ga.

On the night of the 18th of February last, I was sent for by Dr. Wilson Clements, of Sublignа, to aid him in an operation for removal of extensive fragments of fractured skull. The distance being fourteen miles, I did not reach the patient, a little boy three and a half years old, till 8 o'clock the following morning.

Upon examination we found a compound comminuted fracture of the os frontis and parietal bone of the left side of the skull, caused by the kick of a newly shod horse. The detached pieces aggregated two inches in length and a little over one inch in breadth, and were driven in, causing a compression of the brain. The dura mater, arachnoid and pia mater were all ruptured, and disorganized brain material escaped in great quantities, together with considerable discharge of arterial blood from a ruptured branch of the meningeal artery.

The patient was lying in a comatose condition, breathing heavily, with a slow pulse and dilated pupils. We could not mistake the symptoms, and proceeded at once to raise the depressed pieces of bone, three in number, which we found no easy task, as the external table did not break on a line with the internal table, presenting a convexity of the pieces outwards, and we could not get an elevator or tenaculum or anything else under the fragments, and were forced to the necessity of using the trephine, (the common fluted trephine), which we placed upon the edge of the fracture upon the frontal bone, making as small an aperture as possible to serve our purposes. We then succeeded in removing the fragments without further trouble. Upon removing the last and largest fragment of bone a considerable amount of brain substance escaped. During the operation it became necessary to desist from our labor several times, owing to collapse, from which, at one time, it seemed he would not react.

After removing all the fragments of bone, giving time for the exudation of brain and blood, and trimming the rough edges of the ledges of bones, we brought the edges of the wound in the scalp gently together with five sutures; directed cold water applications, low diet, quietude, dark room, and attention to bowels. The patient made a good recovery, in full possession of all his faculties. Furguson, Druit and many others give us remarkable instances of extreme vitality in subjects of skull fracture. The above is another illustration of the fact that extensive fracture of the skull and rupture of membranes and discharge of brain does not in every instance kill.

